# Centering Digital Health Equity During Technology Innovation: Protocol for a Comprehensive Scoping Review of Evidence-Based Tools and Approaches

**DOI:** 10.2196/53855

**Published:** 2024-06-05

**Authors:** Kara Burns, Shoshana Bloom, Cecily Gilbert, Bronwen Merner, Mahima Kalla, Sreshta Sheri, Cleva Villanueva, Amio Matenga Ikihele, Lama Nazer, Raymond Francis Sarmiento, Lindsay Stevens, Ngaree Blow, Wendy Chapman

**Affiliations:** 1 Centre for Digital Transformation of Health University of Melbourne Carlton Australia; 2 Equiti Health UK London United Kingdom; 3 Centre for Health Equity University of Melbourne Carlton Australia; 4 Melbourne Medical School University of Melbourne Carlton Australia; 5 Escuela Superior de Medicina Instituto Politécnico Nacional Mexico City Mexico; 6 Moana Connect Auckland New Zealand; 7 King Hussein Cancer Center Amman Jordan; 8 National Telehealth Center National Institutes of Health University of the Philippines Manila Philippines; 9 School of Medicine Stanford University Palo Alto, CA United States; 10 Medical Education Indigenous Health Faculty of Medicine, Dentistry and Health Sciences University of Melbourne Carlton Australia

**Keywords:** digital health technology, eHealth, mHealth, health informatics, equity, inclusion, participatory design, universal design, Validitron, digital health, cost, technology, technology innovation, innovation, evidence-based tools, evidence-based, tools, digital innovation, cost-effective, accessibility, digital inequity, digital health equity

## Abstract

**Background:**

In the rush to develop health technologies for the COVID-19 pandemic, the unintended consequence of digital health inequity or the inability of priority communities to access, use, and receive equal benefits from digital health technologies was not well examined.

**Objective:**

This scoping review will examine tools and approaches that can be used during digital technology innovation to improve equitable inclusion of priority communities in the development of digital health technologies. The results from this study will provide actionable insights for professionals in health care, health informatics, digital health, and technology development to proactively center equity during innovation.

**Methods:**

Based on the Arksey and O’Malley framework, this scoping review will consider priority communities’ equitable involvement in digital technology innovation. Bibliographic databases in health, medicine, computing, and information sciences will be searched. Retrieved citations will be double screened against the inclusion and exclusion criteria using Covidence (Veritas Health Innovation). Data will be charted using a tailored extraction tool and mapped to a digital health innovation pathway defined by the Centre for eHealth Research roadmap for eHealth technologies. An accompanying narrative synthesis will describe the outcomes in relation to the review’s objectives.

**Results:**

This scoping review is currently in progress. The search of databases and other sources returned a total of 4868 records. After the initial screening of titles and abstracts, 426 studies are undergoing dual full-text review. We are aiming to complete the full-text review stage by May 30, 2024, data extraction in October 2024, and subsequent synthesis in December 2024. Funding was received on October 1, 2023, from the Centre for Health Equity Incubator Grant Scheme, University of Melbourne, Australia.

**Conclusions:**

This paper will identify and recommend a series of validated tools and approaches that can be used by health care stakeholders and IT developers to produce equitable digital health technology across the Centre for eHealth Research roadmap. Identified evidence gaps, possible implications, and further research will be discussed.

**International Registered Report Identifier (IRRID):**

DERR1-10.2196/53855

## Introduction

The United Nations’ Sustainable Development Goals champion a healthful future by ensuring no one is left behind [1]. Achieving health and wellness equity remains a paramount challenge for global health systems [2]. Digital health technologies (DHTs)—encompassing telehealth, electronic health records, mobile devices and apps, wearables, and artificial intelligence (AI)—present a promising solution. Evidence indicates that DHTs are cost-effective for enhancing health outcomes across populations; elevating care quality and convenience; and addressing service accessibility, availability, and capacity concerns [2]. While DHT adoption has increased gradually over the last decade, the COVID-19 pandemic accelerated its integration worldwide across health care, research, government, and industry [3]. However, this digital shift poses a risk to priority communities who may miss out on the technologies and their benefits [4]. Priority communities refer to those who require intentional support due to a history of oppressive policy choices and marginalization. This may be defined by socioeconomic status, race, ethnicity, disability, gender, or other demographic factors. The use of the term “priority community” emphasizes a strengths-based approach that focuses on the need for health system investment to promote equity, ensuring the community is prioritized by that system rather than being made vulnerable by it [5].

Regrettably, priority communities experience “digital exclusion,” or the inability to access digital technologies, and “digital inequity,” which prevents them from effectively engaging with digital technologies to manage their health [6-8]. Evidence shows that priority communities may not have equal access to the underlying technologies that support DHTs, such as mobile phones, computers, and internet services [9-14]. Moreover, communities with culturally or linguistically diverse backgrounds are less likely to have access to culturally appropriate DHTs [15,16]. This was a prominent experience during the COVID-19 pandemic response and vaccination rollout over 2020-2022 [17,18] and demonstrates a need for cultural considerations in health messaging and technologies. Further, priority communities may have concerns about data privacy and lack trust in the digital products and health care systems that provide them [4,13]. In parallel, they are also considered to be underserved by the health system [19], have a greater prevalence of chronic conditions, and experience worse health outcomes [20]. Indeed, they already experience a multitude of factors causing disadvantage, often set against structural barriers like ageism, sexism, racism, and homophobia [21,22]. Thus, digital exclusion and inequity cannot be another mechanism to leave priority communities behind [5].

Poorly designed DHTs that are developed using inaccurate, biased, or incomplete data sets can also worsen health outcomes for priority communities and can further entrench inequities [4,8]. For example, the lack of diverse racial representation during the development of AI health algorithms may introduce negative biases resulting in preferential treatment to some racial groups over others [23]. Data poverty, or a lack of data in low- and middle-income countries, is also a major barrier to the equitable adoption of DHTs with data gaps underrepresenting some populations [22,24]. To counter these disparities, a proactive strategy to center equity is needed by professionals working across health care, health informatics, digital health, and technology development [6-8].

Digital health equity is often defined as equitable access to digital health care and equitable outcomes from digital health care [25], but we argue equity can also be enacted through priority community involvement in the pathway of digital technology innovation. These stages include project contextual inquiry, value specification, design, operationalization, and summative evaluation [26]. When digital health equity is considered along the Centre for eHealth Research (CeHres) roadmap, digital exclusion and inequity may be reduced, and DHTs can even mitigate the preexisting disparity experienced by priority communities [22]. To achieve these outcomes, priority communities must be proactively considered and included throughout the development process [4,27-29].

The factors that drive digital health equity are a growing discourse. Frameworks evolving from health equity typically describe individual, social, and structural factors, or the Digital Determinants of Health. These factors occur on multiple levels affecting equity across the individual, interpersonal, community, and society settings [25,30,31]. Although frameworks and theories are useful guides, it is only through the application of this knowledge to technology innovation and in health care services that an effect on equitable outcomes can be achieved [22,32]. Additionally, the measurement and validation of equity outcomes are topics of debate. A recent systematic review assessing analytical frameworks and economic evaluations of DHTs suggested a lack of equity assessment across multiple assessment domains [33]. Similarly, a rapid review of technologies to address digital health equity found that “limited data are available on the effectiveness of these initiatives in reducing health inequities” [34]. These studies highlight the need to approach equity from an evidence-informed perspective.

To date, a systematically executed review that addresses evidence-based tools and approaches to translate digital health equity appears to be lacking. Hence, this review will chart tools and approaches used during digital health innovation to promote equitable inclusion of priority communities and then identify the gaps at each stage of the CeHRes roadmap [26]. This review builds on previous research identifying inequities and factors that result in the uneven use of DHTs [35] and is distinguished from other scholarly works only covering the design phase in the digital health innovation pathway [36]. Where possible, ideas to explain the identified evidence gaps and the implications of these issues for professionals working across health care, health informatics, digital health, and technology development will arise. Further research will also be identified. Hence, this review will answer the following questions: (1) What tools and approaches are used when involving priority communities in the development of DHTs? (2) Where are these tools and approaches located along the CeHRes roadmap? (3) How is the acceptability of these tools and approaches described by the priority community or measured by researchers?

## Methods

### Participants

Eligible publications will consider priority communities, which may be defined by location (eg, rurality), socioeconomic status, race, ethnicity, disability, older population, gender, children, culture, or other demographic factors.

### Concept

All papers that include approaches or tools for improving digital health equity, defined as equitable inclusion in the development of DHTs [6], will be eligible.

### Context

This scoping review will consider DHT used by priority communities to manage health or access health care services. As articulated by the National Institute for Health and Care Excellence (UK), DHTs include “standalone software and apps that are used to improve health outcomes or to improve how the health and care system runs.” [37] These can include (1) regulated medical devices classed as software as a medical device; (2) software and apps designed to help people to manage their own health and well-being; (3) software that is designed to help the health and care system to run more efficiently or to help staff manage their time, staffing, or resources; and (4) apps or software designed to work alongside a medical device.

To improve the feasibility of the review, traditional imaging that has been transformed into digital imaging and data-driven technologies (blockchain and AI-based algorithms) will be excluded as these are not routinely used in health care services.

The review will consider project contextual inquiry, value specification, design, operationalization, and summative evaluation. Given digital health inequities affect priority communities across high-income and low- and middle-income countries, studies from all countries will be eligible for inclusion in the review.

### Types of Studies

The review will include quantitative studies of experimental and quasi-experimental designs, including peer-reviewed journal articles, relevant to the research question, which meet the inclusion and quality criteria as eligible for inclusion. Study types such as randomized controlled trials, nonrandomized controlled trials, before and after studies, and interrupted time-series studies will be eligible. In addition, analytical observational studies including prospective and retrospective cohort studies, case-control studies, and analytical cross-sectional studies will be considered for inclusion. Descriptive observational study designs including case series, individual case reports, and descriptive cross-sectional studies will also be eligible for inclusion.

Qualitative studies including, but not limited to, designs such as phenomenology, grounded theory, ethnography, qualitative description, action research, and feminist research will also be considered.

Literature reviews will not be included; however, their bibliographies will be hand searched to identify relevant studies. Commentary papers that do not generate empirical evidence will not be eligible. Preprints and theses will also be excluded.

### Ethical Considerations

This work is a literature review of previously published analyses. The University of Melbourne’s Office of Research and Integrity advised that it did not meet the criteria for requiring human ethics review (personal communication, May 17, 2024).

### Review Strategy

This review will be based on the Arksey and O’Malley [38] framework for scoping reviews [39] and will be informed by contemporary guidance from the Joanna Briggs Institute [40]. The 6-stage framework includes identifying research questions; identifying relevant studies; study selection; charting the data; summarizing and reporting the results; and a consultation exercise with groups who are targeted by digital health equity initiatives.

Inclusion and exclusion criteria will be iteratively refined to answer the research question as the scoping review progresses. A 2-stage (title or abstract and full article) screening process will be conducted to choose eligible studies for final inclusion. A standardized data extraction tool will be used to extract and chart the data. Included studies will be analyzed using a qualitative analysis approach summarizing the findings.

### Search Strategy

The search strategy will aim to locate published studies. An initial limited search of MEDLINE and ACM Digital Library was undertaken to identify articles on the topic, from both the health and IT literature. The text words contained in the titles and abstracts of relevant articles and the index terms used to describe the articles were used to develop a full search strategy for the targeted databases (listed in Multimedia Appendix 1). The search strategy, including all identified keywords and index terms, was adapted for each included database or information source. The reference lists of all included sources of evidence will be screened for additional studies. A search is also being conducted for forward citations of key publications identified in this process.

### Inclusion and Exclusion Criteria

Eligible sources for this scoping review will be peer-reviewed papers that report on a tool or approach that has been implemented to promote equitable inclusion during technology innovation. We will include studies published in the English language after 2010. Items in other languages will be included if they include an English abstract that clearly reports the required data. The authors of relevant studies will also be contacted where necessary for study details. Items for which full text is not available will be excluded.

### Evidence Selection

Following the search, all identified citations will be collated and uploaded into Covidence (Veritas Health Innovation) for the removal of duplicates and management of the subsequent stages of the review screening and data extraction processes [41]. After the pilot screening test of 10% of the citations, 1 reviewer will screen the items in the title or abstract stage, against the inclusion criteria for the review. Potentially relevant sources will be retrieved in full. The full text of selected citations will be assessed in detail against the inclusion criteria by 2 or more independent reviewers. The reasons for the exclusion of sources of evidence at the full-text stage that do not meet the inclusion criteria will be recorded and reported in the scoping review. Any disagreements that arise between the reviewers at each stage of the selection process will be resolved through discussion or with an additional reviewer. The results of the search and the study inclusion process will be reported in full in the final scoping review and presented in a PRISMA-ScR (Preferred Reporting Items for Systematic Reviews and Meta-Analyses Extension for Scoping Reviews) flow diagram [42]*.*

### Data Extraction

Data will be extracted from papers by 2 independent reviewers using a data extraction tool developed for the project and piloted by authors across multiple fields of research (Textbox 1). The data extracted will include specific details about the participants, concept, context, study methods, and key findings relevant to the review questions and mapped to the digital innovation pathway through the CeHRes roadmap for the development of eHealth technologies [26].

**Data extraction form.**

- Authors
- Year of publication
- Country of study
- Aim of the research
- Methodology (study design, interventions, description, and analysis techniques)
- Population
- Concept
- Context
- Low-, middle-, or high-income country
- Identified barriers to achieving digital health equity
- Identified facilitators for achieving digital health equity
- Tool description
- Use of theory to inform paper or tool development (if available)
- Stakeholder (eg, patients, end users, and priority communities) involvement in the tool co-design
- Results
- Authors’ recommendations
- Relevant stage of the Centre for eHealth Research roadmap

### Data Analysis and Presentation

Data will be deductively mapped to the CeHRes roadmap for the development of eHealth technologies [26]. The roadmap includes 5 stages; however, some technologies may be distributed across multiple stages, and emergent stages may also be discovered:

1. Contextual inquiry—this stage involves gathering information from intended users and relevant stakeholders about the nature of the problem and potential solutions.
2. Value specification—this stage involves determining and ranking the social, economic, medical, and behavioral values of key stakeholders. From this process, the most favorable solutions are identified, as well as the user and organizational requirements to achieve them.
3. Design—this stage involves developing prototypes that align with the values and user requirements.
4. Operationalization—this stage involves the implementation of the technology into practice. Operationalization may include enabling activities, training, education, and deploying the technology into practice.
5. Summative evaluation—this stage refers to the actual uptake of the technology in practice and its clinical, organizational, and behavioral impacts.

A narrative summary will accompany the data map to describe how the results relate to the review objectives and research question.

## Results

Screening of records retrieved from databases and other sources has reached the full-text stage and is expected to be complete by the end of May 2024. Data extraction (mapping to the CeHRes roadmap stages) and synthesis are scheduled between June and December 2024. Following a planned exposure of the review’s findings to our panel of stakeholders including consumers and digital health innovation experts, we will disseminate the final review findings in a peer-reviewed journal publication and presentations at relevant conferences.

## Discussion

### Principal Findings

Noting the apparent absence of an overarching review of evidence-based tools and methods to translate digital health equity in all stages of digital health innovation, our scoping review will identify these tools and methods and map them to stages in the CeHRes roadmap (contextual inquiry, value specification, design, operationalization, and summative evaluation). Our work is aimed at synthesizing how priority communities initiate or are brought into the development and evaluation of DHTs and how they describe the tool’s acceptability. We will also note how researchers measure the acceptability of the tools or approaches used.

### Limitations

We may encounter limitations in the published literature that prevent us from building a factual account of the range of methods or tools that have been trialed with priority groups. In particular, there might be an absence of reports of negative initiatives or tools that failed to achieve equity outcomes, since positive study results are more likely to be published [43]. We acknowledge that excluding non-English language studies may bias the outcomes we are able to include. Additional research using material in data repositories and non-English studies could remedy these issues.

### Conclusions

The scoping review will identify tools and approaches that have been validated for use by health care technology developers to enable equitable digital innovations. Where gaps are detected in either the available evidence or in their span across the 5 stages of the CeHRes roadmap, these will be highlighted, and their possible implications will be proposed. The work described in this protocol supports the methodological development of the Digital Health Validitron, a collaborative and interdisciplinary research group that assists digital health innovators from health care, academia, and industry to accelerate the creation of evidence that proves the real-world value of their ideas and products.

## Appendix

Multimedia Appendix 1 Search strategy.

## Abbreviations

AI: artificial intelligence
CeHRes: Centre for eHealth Research
DHT: digital health technology
PRISMA-ScR: Preferred Reporting Items for Systematic Reviews and Meta-Analyses Extension for Scoping Review
