# Supplementary material for: Centering Digital Health Equity During Technology Innovation: Protocol for a Comprehensive Scoping Review of Evidence-Based Tools and Approaches
Source: JMIR Res Protoc. 2024 Jun 5;13:e53855. doi: 10.2196/53855 (PMC11187514; doi:10.2196/53855)
Supplement: Multimedia Appendix 1 [file resprot_v13i1e53855_app1.docx]

***Databases to be searched:***

| **Computing, Information Science and Systems** | |
| --- | --- |
| ACM Digital Library Complete | On ACM platform |
| Compendex | On Elsevier’s *Engineering Village* platform |
| IEEE Xplore Journals | On IEEE platform |
| Inspec | On Elsevier’s *Engineering Village* platform |
| **Health and Medicine databases** | |
| Medline | On OvidSP platform |
| CINAHL *Cumulative Index to Nursing and Allied Health Literature* | On EBSCOHost platform |
| PsycInfo | On OvidSP platform |
| **Multidisciplinary aggregations** | |
| ProQuest One Academic | On ProQuest platform. |
| Web of Science | On Clarivate platform. |
| Dimensions | On Digital Science platform. |

***Sample Database Search Strategy***

Ovid MEDLINE(R) ALL <1946 to November 09, 2023>

1 exp Healthcare Disparities/ 22454

2 exp Health Status Disparities/ 19934

3 (disparit* or discrimina* or equit* or inequit* or marginali* or underserved).tw,kw,kf. 514767

4 ((cultur* or ethnic* or gender* or racial or relig*) adj3 minorit*).tw,kw,kf. 25120

5 exp *Socioeconomic Factors/ 184351

6 (socio-economic adj disadvantage*).tw,kw,kf. 540

7 exp Digital Divide/ 192

8 Health Literacy/ 9472

9 ((digital* adj literate) or (digital adj literac*)).tw,kw,kf. 818

10 Health Services for Persons with Disabilities/ 149

11 exp *Disabled Persons/ 58126

12 ((disabl* or impair*) adj3 (people* or person* or individual* or child* or youth* or population* or worker* or men or women or man or woman or communit* or physical*)).tw,kw,kf. 53560

13 exp Rural Health Services/ 14158

14 Rural Health/ or *Rural Population/ 45735

15 ((rural or remote) adj3 (population* or communit* or people* or youth* or patient*)).tw,kw,kf. 51248

16 exp Digital Technology/ 778

17 exp Electronic Health Records/ 28323

18 mobile applications/ 11871

19 telemedicine/ or telehealth/ 38249

20 informatics/ or exp consumer health informatics/ or exp dental informatics/ or exp medical informatics/ or exp nursing informatics/ or exp public health informatics/ 504649

21 (digital adj health).tw,kw,kf. 8477

22 (ehealth or e-health or eportal* or e-portal* or mhealth or m-health or (patient adj5 portal*)).tw,kw,kf. 24197

23 (telecare or tele-care or teleconsult* or tele-consult* or telehealth* or tele-health* or telemedicine or tele-medicine).tw,kw,kf. 37938

24 (tool* or framework* or resource* or roadmap* or schema).tw,kw,kf. 1818692

25 or/1-15 900353

26 or/16-23 601218

27 25 and 26 34122

28 24 and 27 8263

29 (validat* or evaluat*).tw,kw,kf. 5057724

30 28 and 29 3409

31 limit 30 to (english language and yr="2010 -Current") 2887

[END]
